# Shynthesis and Characterizations of Calcium Hydroxyapatite Derived from Crabs Shells (*Portunus pelagicus*) and Its Potency in Safeguard against to Dental Demineralizations

**DOI:** 10.1155/2015/469176

**Published:** 2015-07-07

**Authors:** Indah Raya, Erna Mayasari, Afdaliah Yahya, Muhammad Syahrul, Andi Ilham Latunra

**Affiliations:** ^1^Chemistry Department, Faculty of Mathematics and Natural Sciences, Hasanuddin University, Makassar 90245, Indonesia; ^2^Biology Department, Faculty of Mathematics and Natural Sciences, Hasanuddin University, Makassar 90245, Indonesia

## Abstract

Crab's shells of Portunus pelagicus species were used as raw materials for synthesis of hydroxyapatite were used for protection against demineralization of teeth. Calcination was conducted to crab's shells of Portunus pelagicus at temperature of 1000°C for 5 hours. The results of calcination was reacted with (NH_4_)_2_HPO_4_, then dried at 110°C for 5 hours. Sintering was conducted to results of precipitated dried with temperature variations 400–1000°C for a hour each variation of temperature then characterized by X-ray diffractometer and FTIR in order to obtain the optimum formation temperature of hydroxyapatite is 800°C. The hydroxyapatite is then tested its effectiveness in protection against tooth demineralization using acetate buffer pH 5.0 with 1 M acetic acid concentration with the addition of hydroxyapatite and time variation of immersion. The results showed that the rate of tooth demineralization in acetate buffer decreased significantly with the provision of hydroxyapatite into a solution where the addition of the magnitude of hydroxyapatite is greater decrease in the rate of tooth demineralization.

## 1. Introduction

Hydroxyapatite biomaterials are materials that are very widely used in several health purposes, including as a source of calcium for the manufacture of toothpaste and as an important material in the formation/bone repair. The chemical properties of hydroxyapatite are bioactive and compatible with the adjacent bone and teeth. Hydroxyapatite is a calcium phosphate ceramic that is totally biocompatible and nontoxic and becomes an integral part of living bone and teeth tissue [1–8]. So it is important that these materials are produced independently. The raw material for the production of hydroxyapatite biomaterial is very easily available and abundant in Indonesia. The production process was easy and the cost is also relatively inexpensive if done on a large scale. Among the abundant raw materials are the shells of crabs, which are one of Indonesia's main export commodities. Export of Crabs commodity by Indonesia amounted to 604.215–625.000 tons/year without the shell form, while domestic consumption is expected to be so much more, as in Makassar, carrying 292.5 tons exported in the form of crab without shell with the main export destination being Singapore [9]. If the mass of crab shells 25–50% of the total mass, it can be estimated that in 2012 produced the shells of crabs around 151,053.75 to 302,107.5 tons in Indonesia and 73.125 to 146.25 tons in Makassar (a region of Indonesia), there was just from crabs production were exported. This value is of course even more if the crab's consumptions in the country are also taken into account. This suggests the existence of crab shells is abundant in Indonesia, including in Makassar. As known in Indonesia, the shells of crabs have not been used, so it will only be a waste disturbing environment [7, 8].

Crabs shells containing calcium carbonate (CaCO_3_) are very abundant; amount 40–70%, varies according to the species [7]. Calcium carbonate can be further processed into calcium hydroxyapatite [Ca_10_(PO_4_)_6_(OH)_2_] [4, 5]. According to Strassler [10], hydroxyapatite is one of the active ingredient materials that are widely used in toothpaste products for protection against teeth demineralization [11–13].

The crystals structure of hydroxyapatite will be better by using CaO as a precursor of calcium. However, the use of these compounds also produces carbonate apatite [Ca_10_(PO_4_)_6_CO_3_] in a fairly large percentage. This is because the calcination process cannot completely eliminate carbon dioxide (CO_2_) in CaCO_3_ so that there can be reaction with the precursor phosphate. However, the carbonate apatite heating at a temperature of 700°–900°C for 2–5 hours, followed by washing using distilled water, the carbonate apatite can be hydroxyapatite [11–13]. The instrument were used in this research were glass apparatus, Ohaus analytical balance, petri dish, porcelain cup, Buchner flask, Buchner funnel, vacuum pump Sargent-Welch Co. Model 1400, magnetic stirrer, magnetic bar, hotplate Idealife, pH meter, Furnace Thermolyse 6000-Barnstead, dessicator, thermometers, Spnisosfd oven, stopwatch, Shimadzu X-ray diffractometer (XRD) Model 6000, X-ray Fluorescence (XRF) Shimadzu, Fourier Transform Infra-Red (FTIR) Prestige-21 Shimadzu, Scanning Electron Microscopy combined with the ability to generate localized chemical information (SEM-EDXA) Variant, and UV-VIS Shimadzu model 6105. This is because the carbonate ion compounds are able to inhibit the crystallization process Ca_10_(PO_4_)_6_(OH)_2_ so that the results will be dominated by an amorphous phase [14].

The calcinations temperatures of CaCO_3_ range from 900° to 1200°C. If the CaCO_3_ burned at temperature calcinations, decomposition reaction of CaCO_3_ into CaO will occur and CO_2_ emissions are dominant and will be issued as a result of the combustion reaction [13–15].

## 2. Experimental

### 2.1. Research Materials

The following materials used are as follows. Waste of crabs shells* Portunus pelagicus *species was taken from an exporter crabs company in industry areal, Makassar city, south Sulawesi province, Indonesia. Tooth samples were taken from Dr. Wahidin hospital, and (NH_4_)_2_HPO_4_ was obtained from Fluka chemical, CH_3_COOH glacial 100%, CH_3_COONa·3H_2_O from Merck, (NH_4_)_6_Mo_7_O_24_·4H_2_O from Aldrich, NH_4_VO_3_ from Aldrich, HNO_3_ and NaF from Fluka chem., (NH_4_)_2_C_2_O_4_, distilled water, aluminum foil, and Whatman filter paper numbers 40 and 42.

### 2.2. Research Instruments

The instruments were used in this research that glass as commonly used in laboratories, Ohaus analytical balance, petri dish, porcelain cup, Buchner flask, Buchner funnel, vacuum pump Sargent-Welch Co. Model 1400, magnetic stirrer, magnetic bar, hotplate Idealife, pH meter, Furnace Thermolyse 6000 Barnstead, dessicator, thermometers, oven Spnisosfd, stopwatch, Shimadzu X-ray diffractometer (XRD) Model 6000, X-ray fluorescence (XRF) Shimadzu, Fourier transform infrared (FTIR) Prestige-21 Shimadzu, scanning electron microscopy combined with the ability to generate localized chemical information (SEM-EDXA) variant, and UV-VIS Shimadzu model 6105.

### 2.3. Synthesis of Hydroxyapatite from Waste Shell Crab* Portunus pelagicus*

Crabs shells* Portunus pelagicus *waste was cleaned with distilled water and dried at room temperature. Furthermore, to transform crabs shells into CaCO_3_ and then into CaO, calcinations were performed on the samples at 1000°C temperature for 5 hours at a rate of temperature rise 5°C/minute. The containment of calcium (Ca) was determined by using XRF. Calcium oxide (CaO) obtaining dominated as result of calcinations and then made suspensions in 100 mL of distilled water with a calcium concentration of 0.3 M. The suspensions reacted by dropwise with a 100 mL 0.2 M of (NH_4_)_2_HPO_4_ solution through the coprecipitation method, at temperatures around 40°C while the solution was stirred for 2–5 hours. The precipitation allowed stand overnight or 24 hours at room temperature, and the precipitate is filtered with a Whatman filter paper number 40 and dried at 110°C for 5 hours. The pure hydroxyapatite obtained by sintering to the dried precipitate at various temperatures of 500°–900°C for 4 hours [16, 17]. The results washed with distilled water and then dried at a temperature of 110°C. The characterization of the compounds was performed by using X-ray diffraction (XRD), FTIR, and SEM-EDXA.

### 2.4. The Teeth Demineralization Tested

The proven in vitro demineralization of teeth was conducted through evaluating the concentration of phosphate in solution by using the UV-Vis spectroscopy. The effectiveness of hydroxyapatite to protection against teeth demineralization was tested in acetate buffer pH 5.0 solutions, with 1 M of acetic acid concentration with the addition of hydroxyapatite in varying concentrations and immersion time [18]. Each of 5 beakers was filled with 300 mL of acetate buffer pH 5.0 with acetic acid concentration of 1 M. An acetate buffer was left without adding anything as a comparison. An acetate buffer was then added 10 ppm of NaF left without addition of Ca_10_(PO_4_)_6_(OH)_2_. Other three pieces beaker of acetate buffer containing 10 ppm of NaF are adding of Ca_10_(PO_4_)_6_(OH)_2_ with variation concentration of 25 ppm, 50 ppm, and 100 ppm. The cleaned tooth samples were immersed in each of 5 beakers of solutions. The immersed times of tooth samples in solution are 3, 6, 9, 24, and 48 hours, respectively. Furthermore, the phosphate concentration in each solution was measured by using UV-Vis spectrophotometer with the wavelength for phosphate (*λ*maks) being 432 nm.

## 3. Results and Discussion

### 3.1. Calcinations of Crab Shells

**Figure 1:**
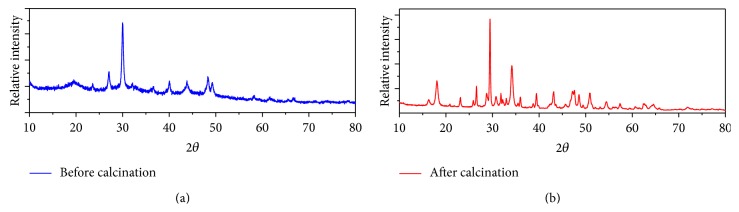
Diffractogram of crab shell; (a) before calcinations and (b) after calcinations at temperature 1000°C for 5 hours.

Synthesis Ca_10_(PO_4_)_6_(OH)_2_ begins with a calcinations crab shell at 1000°C for 5 hours. The calcinations aim to eliminate the organic component in the shells of crabs and convert CaCO_3_ compound which is the dominant compound in the shells of crabs into CaO through the elimination of CO_2_ in gas form. Characterizing the calcinations results was conducted by X-ray, XRF, and FT-IR. The results can be seen from the diffraction pattern of the crab shells before and after calcinations at a temperature of 1000°C for 5 hours (Figure 1). In this figure can be observed changes in the diffraction pattern of crab shells, where the change in the diffraction pattern is due to the chemical change from mixture of CaCO_3_ with organic matter to pure CaO. The appearance of the sharper peaks in Figure 1 after calcinations (b) is a result of the crystallinity of the CaO.

**Figure 2:**
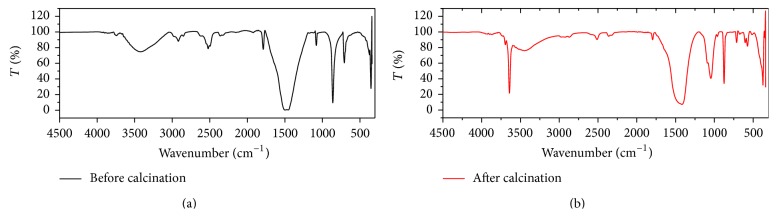
FTIR spectra of crab shells (a) before calcinations and (b) after calcinations at a temperature 1000°C for 5 hours.

Identification by FT-IR as shown in Figure 2 showed there is a reduction process of -CO_3_ groups and some of IR-spectra were missing after calcinations. This shows the elimination of CO_2_ and organic components occurred [19]. The elimination of -CO_3_ groups and organic components can also be seen from the data of mass reduction of sample calcinations. Mass reduction during the calcinations process is 56.35% on average. This means that the efficiency of calcium compounds produced by 43.64%.

The determinations of calcium contained in sample was conducted by using X-ray fluorescence, where obtained calcium is 66.62% after calcinations. These results are then used to calculate the stoichiometry in determining the number of results calcinations which is needed to react with (NH_4_)_2_HPO_4_ as the precursor phosphate.

### 3.2. Precipitation with Phosphate Precursors

The precipitation reactions aiming to produce Ca_10_(PO_4_)_6_(OH)_2_ used phosphate, (NH_4_)_2_HPO_4_, as the precursor and then reacted with CaO as calcinations results. Side results Ca_10_(PO_4_)_6_CO_3_ also occur as a product of reaction of (NH_4_)_2_HPO_4_ and CaCO_3_ presence in the calcinations results.

Dried precipitate further sintered on temperature variations of 400°–1000°C for 2 hours; it is intended to determine the optimum temperature where the Ca_10_(PO_4_)_6_(OH)_2_ is formed, furthermore characterized by using XRD, FT-IR, and SEM-EDXA.

### 3.3. Characterization of Sintering Results by XRD

**Figure 3:**
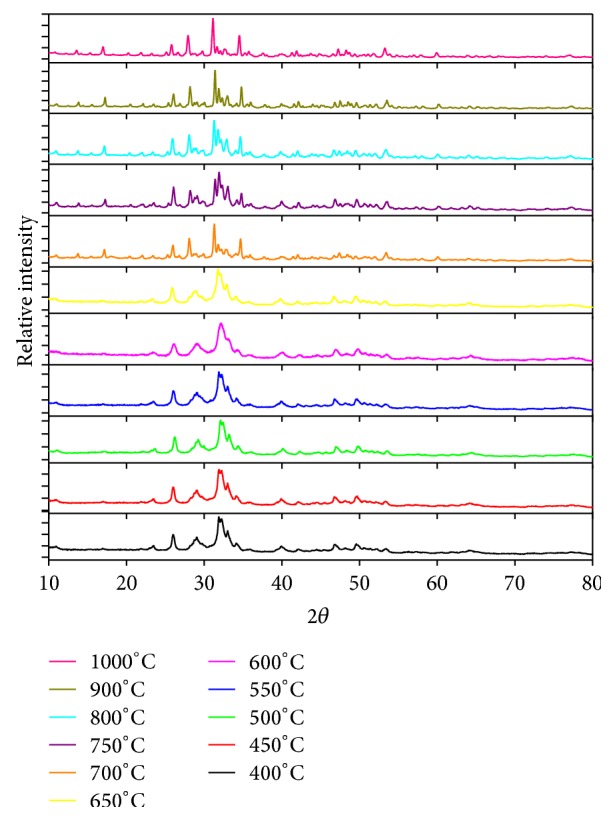
Diffraction patterns as a function of temperature sintering of hydroxyapatite.

XRD Diffractograms of compounds results presented in Figure 3. The diffractograms of each sintering results compounds indicate that the temperature is closely related to the formation of crystals. This is due to the nature of the vibrating atoms moving faster in higher temperatures [19].

**Figure 4:**
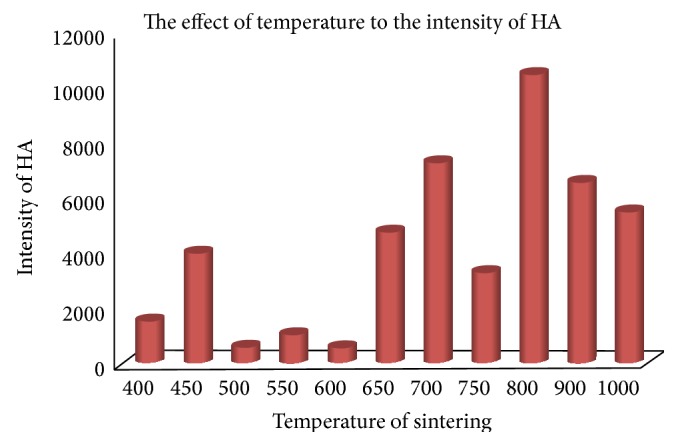
The influence of sintering temperatures on the formation of hydroxyapatite phase.

The optimum temperature formation of hydroxyapatite was determined by calculation of the probability of the sample phase from the XRD results analysis according to JCPDS standard data, which, JCPDS; 24-0033 is standard data for Ca_10_(PO_4_)_6_(OH)_2_; 09-0169 for *β*-Ca_3_(PO_4_)_2_; 29-0359 for *α*-Ca_3_(PO_4_)_2_, 35-0180 *α*-Ca_3_(PO_4_)_2_; 35-0180 for Ca_10_(PO_4_)_6_CO_3_(OH)_2_ and 19-0272 standard data for Ca_10_(PO_4_)_6_CO_3_(OH)_2_. Figures 3 and 4 showed that the temperature is closely associated with the formation of hydroxyapatite phase. In the both graphs it can be seen that the maximum intensity of the phase formation of hydroxyapatite has been found by sintering at temperature of 800°C, it means the optimum temperature of hydroxyapatite formation is 800°C, and then this result will be used for another application.

**Figure 5:**
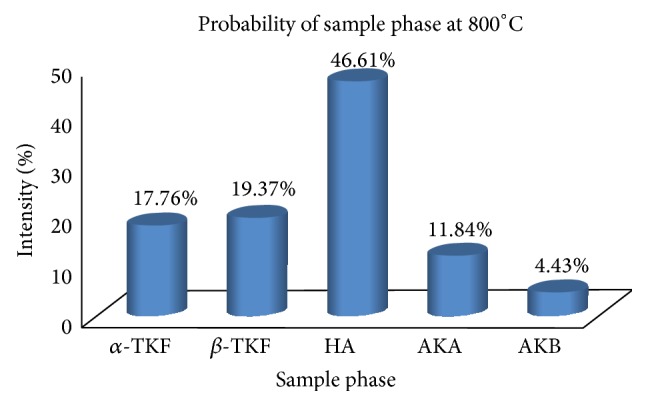
Percentage probability sample phase sintering at a temperature 800°C, where HA = Ca_10_(PO_4_)_6_(OH)_2_, *α*-TKF = *α*-Ca_3_(PO_4_)_2_, *β*-TKF = *β*Ca_3_(PO_4_)_2_, AKA = Ca_10_(PO_4_)_6_CO_3_ and AKB = Ca_10_(PO_4_)_6_CO_3_(OH)_2_.

The formations of hydroxyapatite phase had been dominated at 800°C confirmed by percentage probability sample phase (Figure 5), in which the percentage of hydroxyapatite phase formed around 46.61%, while phases of *α*-Ca_3_(PO_4_)_2_ and phase *β*-Ca_3_(PO_4_)_2_ are 17.76% and 19.37%, respectively. However, also there is still presence of a phase Ca_10_(PO_4_)_6_CO_3_ and Ca_10_(PO_4_)_6_CO_3_(OH)_2_ with a range of 11.84% and 4.43%, respectively, which indicates the presence of carbonates. All data coming from the calculations of the XRD spectrum used its software.

**Figure 6:**
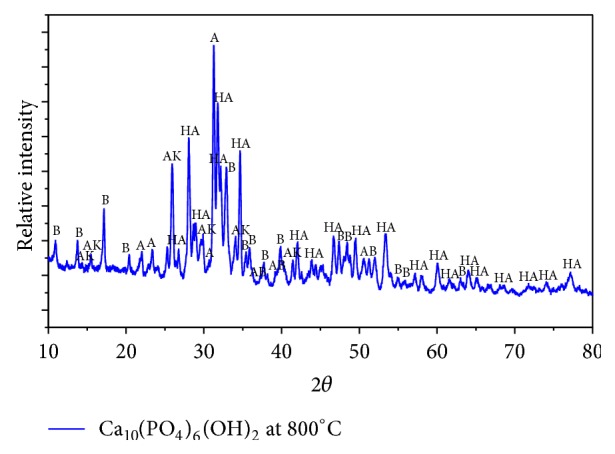
Diffractogram of sample were sintered at 800°C, where, HA = Ca_10_(PO_4_)_6_(OH)_2_, A= *α*-TKF = *α*-Ca_3_(PO_4_)_2_, B = *β*-TKF = *β*Ca_3_(PO_4_)_2_, AK = AKA = Ca_10_(PO_4_)_6_CO_3_ and AB = AKB = Ca_10_(PO_4_)_6_CO_3_(OH)_2_.

The X-ray diffractograms of Ca_10_(PO_4_)_6_(OH)_2_ synthesized at a 800°C temperature sintering can be seen in Figure 6, where the peaks of HA are symbolized by the peak of the crystal Ca_10_(PO_4_)_6_(OH)_2_, while peak *α*-TKF is symbolized crystalline peaks of *α*-Ca_3_(PO_4_)_2_, the symbol *β*-TKF for crystalline *β*-Ca_3_(PO_4_)_2_, AKA for Ca_10_(PO_4_)_6_CO_3_, and AKB for Ca_10_(PO_4_)_6_CO_3_(OH)_2_. The highest intensity peak at 31.2572 deg corresponding to crystalline *α*-Ca_3_(PO_4_)_2_ is seen, the second highest peak intensity is 31.7783, and the third highest peak intensity is 28.0565 crystals suitable for Ca_10_(PO_4_)_6_(OH)_2_.

### 3.4. Characterization of Ca-Hydroxyapatite with FT-IR

**Figure 7:**
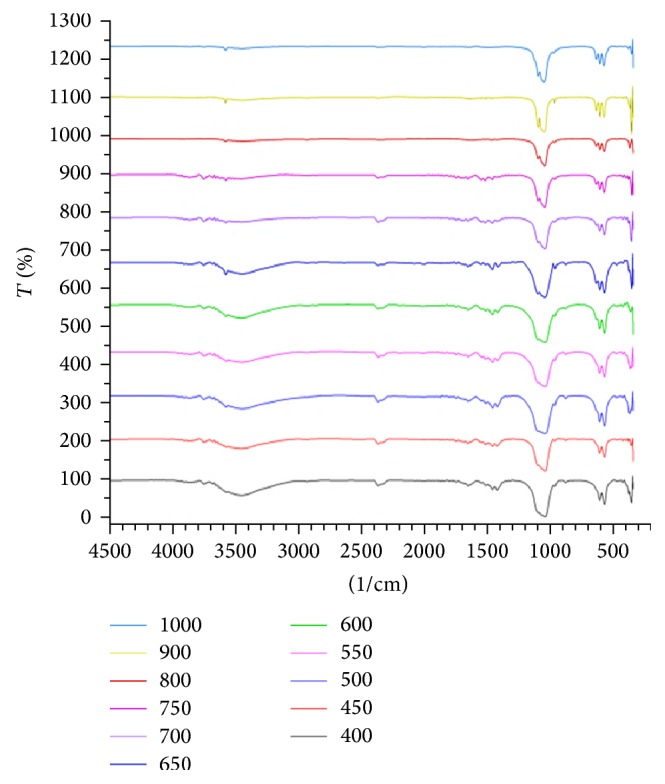
FT-IR spectra of synthesis results in various temperatures (400–1000°C).

FTIR results showed that the sintering temperature variation affects the absorption band shapes which generally all sintering results showed absorption band of -OH, absorption band *υ*1, *υ*2, *υ*3, and *υ*4 of PO_4_^3−^, and CO_3_^2−^ groups. Infrared spectra in Figure 7 show the -OH groups at 633 cm^−1^ which are characteristic of hydroxyapatite [17] appearing on the sintering temperatures of 400–1000°C. Additionally spectrum also showed higher sintering temperature causing the sharper peaks phosphate group (PO_4_^3−^) because the nature of the vibrating atoms moves faster at higher temperatures [19]. The presence of the phosphate group indicates the formation of hydroxyapatite in the precipitates.

**Figure 8:**
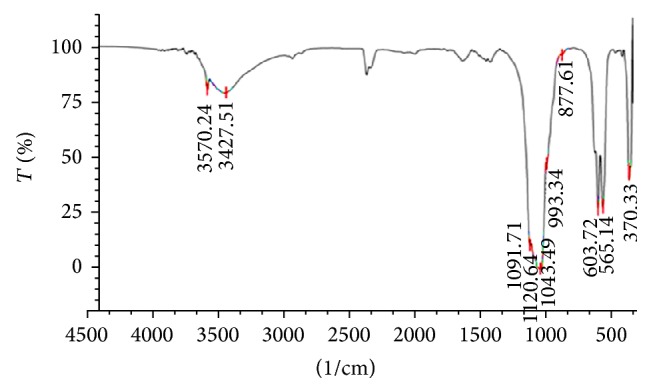
FT-IR spectra of hydroxyapatite (HA) were sintered at 800°C.

The FT-IR spectra of sample were sintered at a temperature of 800°C (Figure 8) showing that hydroxyapatite is the dominant compound formed. The stretching frequencies of PO_4_ group are indicated by 1120.64 cm^−1^, 1091.71 cm^−1^, 1043.49 cm^−1^ (*υ*3), 993.34 cm^−1^, 877.61 cm^−1^ (*υ*1), 603.72 cm^−1^, 565.14 cm^−1^, (*υ*4), and 370.33 cm^−1^ (*υ*_2_). And a sharp spectrum in the area of 3570.24 cm^−1^ indicates the presence of free -OH and 3427.51 cm^−1^ indicating -OH bounded, and this is indicating that the dominant compound is Ca_10_(PO_4_)_6_(OH)_2_. While area of 1654.92 cm^−1^, 1458.18 cm^−1^, and 1421.54 cm^−1^ indicates the presence of carbonate groups (CO_3_^−^) it can be identified as Ca_10_(PO_4_)_6_CO_3_ and Ca_10_(PO_4_)_6_CO_3_(OH)_2_ which has not been transformed into Ca_10_(PO_4_)_6_(OH)_2_ during the sintering process.

### 3.5. Characterization of Ca-Hydroxyapatite Sintered at 800°C with SEM-EDXA

**Figure 9:**
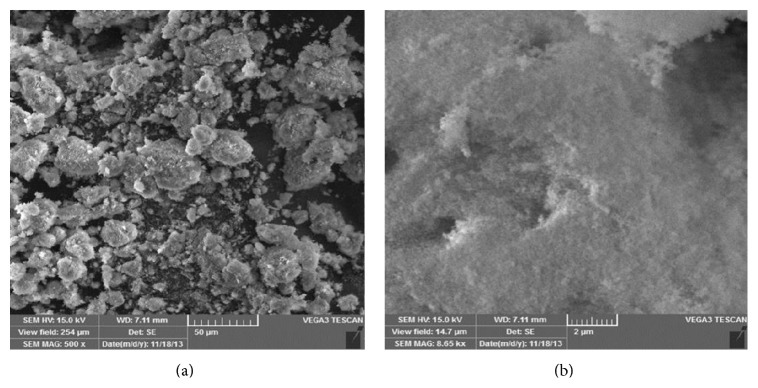
The SEM graph of hydroxyapatite (HA) synthesis from crab shells.

The results of characterization by SEM-EDXA shown in Figure 9(a) show that the size of hydroxyapatite formed from the synthesis tends to be small and only a few are large. While Figure 9(b) shows that the surface of hydroxyapatite is smooth and nonporous, this shows that hydroxyapatite which has synthesis from crab shells can function well as inhibiting tooth demineralization [13].

**Figure 10:**
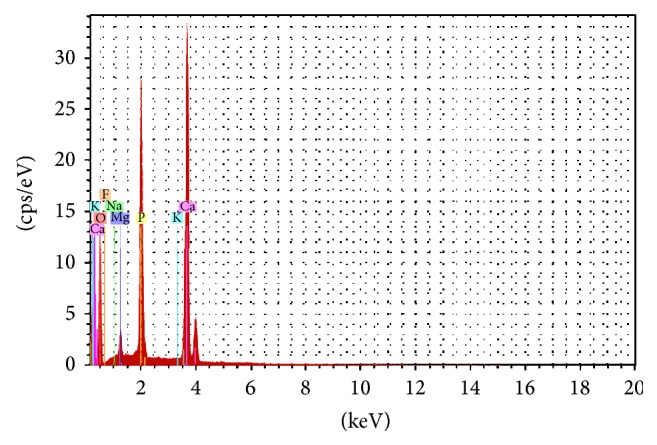
The EDXA spectrum of hydroxyapatite (HA) synthesis from crab shells.

While in Figure 10 the EDXA spectrum shows the composition of the synthesis yield was dominated by oxygen (O) up to 59.52%, calcium (Ca) up to 23.76%, and phosphorus (P) up to 13.32%. The composition confirmed the composition of hydroxyapatite. It can be concluded that the synthesis results can be achieved to target.

### 3.6. Inhibition of Tooth: The Demineralization by Presence of Ca_10_(PO_4_)_6_(OH)_2_

Demineralization of teeth is a process of decomposition of the crystal of Ca_10_(PO_4_)_6_(OH)_2_ due to the acidic conditions by releasing Ca^2+^ and PO_4_^3−^ ions. Demineralization of tooth causing increased levels of Ca^2+^ and PO_4_^3−^ in saliva in direct contact with the tooth. In vitro, the rate of tooth demineralization can be observed through the concentrations of Ca^2+^ and PO_4_^3−^ ions in solutions where the tooth was soaked each unit of time. Therefore, increase of PO_4_^3−^ ions concentrations in solution a soaked gear can be one of indicators to measure the rate of tooth demineralization.

**Figure 11:**
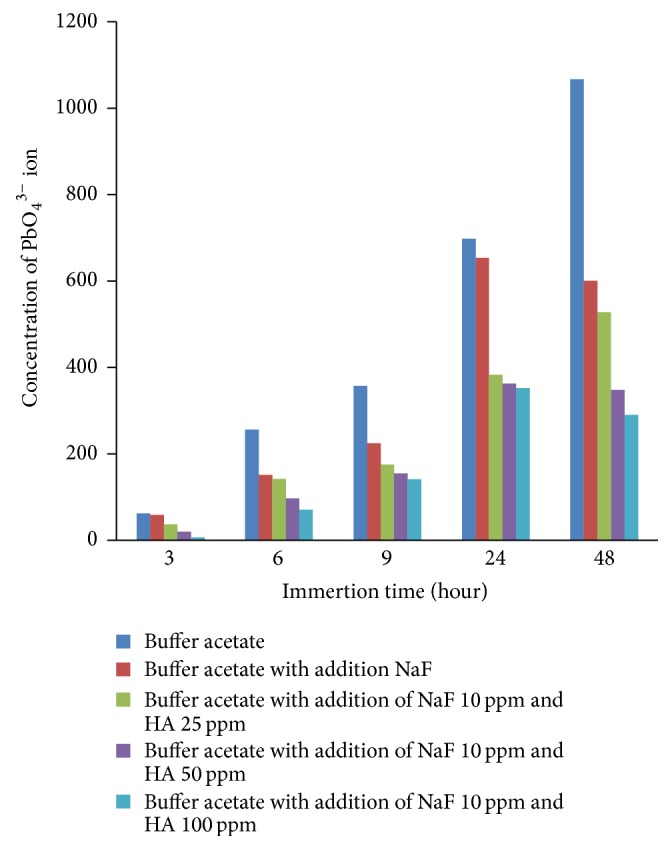
Relationship between teeth immersion time versus PO_4_^3−^ ion concentration in a solution where teeth soaked or immersed.

Figure 11 shows the relationship between the soaking time of teeth versus the increase of the ion PO_4_^3−^ levels in solution where the tooth was soaked as well; it appears that with the increasing addition of Ca_10_(PO_4_)_6_(OH)_2_ into the acetate buffer equals to the rate of demineralization decrease. It is can be altered by the amount of PO_4_^3−^ ions in solutions; however the addition of Ca_10_(PO_4_)_6_(OH)_2_ ions showed lower amount of PO_4_^3−^ ions compared to solutions without the addition of Ca_10_(PO_4_)_6_(OH)_2_. This proves that the Ca_10_(PO_4_)_6_(OH)_2_ were synthesized from the crab shell effective for protection against tooth demineralization.

The decrease in the rate of tooth demineralization with the addition of Ca_10_(PO_4_)_6_(OH)_2_ can also be observed through analyzing the tooth mass reduction in the fifth variation of acetate buffer solution as shown in Table 1. The greater concentrations of Ca_10_(PO_4_)_6_(OH)_2_ in the acetate buffer solution were teeth immersed exhibit the smaller mass of teeth in the solution [19].

Table 1 shows the relationship between tooth mass and the addition of hydroxyapatite.

## 4. Conclusion

Based on these results it is concluded as follows.

1. Waste of shells crabs (*Portunus pelagicus*) proved to be used as raw material for the synthesis of Ca_10_(PO_4_)_6_(OH)_2_ due to high calcium levels which amounted to 66.62% in addition to the abundant existence as waste.
2. The optimum temperature formation of Ca_10_(PO_4_)_6_(OH)_2_ is at 800°C.
3. Ca_10_(PO_4_)_6_(OH)_2_ were synthesized from waste of shell crabs (*Portunus pelagicus*) in vitro effectively inhibiting the rate of demineralization of the tooth where the greater the addition of Ca_10_(PO_4_)_6_(OH)_2_ in the solution, the more the inhibiting demineralization of the tooth or the smaller the rate of tooth demineralization in solution.

## Figures and Tables

**Table 1:** Percentage teeth mass remaining on immersion in acetate buffer solution with a certain variation of the treatment for 48 hours.

Number	Code sample		Initial mass (gram)	Final mass (gram)	Percentage (%)
1	TP	G 1	1,54	1,52	98,21
		G 2	1,07	1,04	97,81
		G 3	1,73	1,69	97,55
		Total			**97,86**

2	P	G 1	0,79	0,77	96,96
		G 2	0,82	0,79	96,85
		G 3	1,17	1,15	97,99
		Total			**97,27**

3	25	G 1	1,97	1,92	97,74
		G 2	1,23	1,21	98,84
		G 3	1,65	1,62	97,81
		Total			**98,13**

4	50	G 1	0,91	0,89	98,33
		G 2	1,20	1,18	98,01
		G 3	1,26	1,24	98,10
		Total			**98,15**

5	100	G 1	1,4589	1,44	98,70
		G 2	1,053	1,0307	97,88
		G 3	1,4709	1,4448	98,22
		Total			**98,27**

G = teeth.

TP = acetate buffer without the addition of any NaF and Ca_10_(PO_4_)_6_(OH)_2_.

P = acetate buffer with only the addition of NaF.

25 = acetate buffer addition of NaF and 25 ppm of Ca_10_(PO_4_)_6_(OH)_2_.

50 = acetate buffer addition of NaF and 50 ppm of Ca_10_(PO_4_)_6_(OH)_2_.

100 = acetate buffer addition of NaF and 100 ppm of Ca_10_(PO_4_)_6_(OH)_2_.
